# A Recurrent Neural-Network-Based Real-Time Dynamic Model for Soft Continuum Manipulators

**DOI:** 10.3389/frobt.2021.631303

**Published:** 2021-03-18

**Authors:** Abbas Tariverdi, Venkatasubramanian Kalpathy Venkiteswaran, Michiel Richter, Ole J. Elle, Jim Tørresen, Kim Mathiassen, Sarthak Misra, Ørjan G. Martinsen

**Affiliations:** ^1^ Department of Physics, University of Oslo, Oslo, Norway; ^2^ Department of Biomechanical Engineering, University of Twente, Enschede, Netherlands; ^3^ The Intervention Centre, Oslo University Hospital, Oslo, Norway; ^4^ Department of Informatics, University of Oslo, Oslo, Norway; ^5^ Department of Technology Systems, University of Oslo, Oslo, Norway; ^6^ Department of Biomedical Engineering, University of Groningen and University Medical Centre Groningen, Groningen, Netherlands; ^7^ Department of Clinical and Biomedical Engineering, Oslo University Hospital, Oslo, Norway

**Keywords:** continuum manipulators, soft robotics, dynamic models, Cosserat rod theory, Lie group variational integration, recurrent neural network

## Abstract

This paper introduces and validates a real-time dynamic predictive model based on a neural network approach for soft continuum manipulators. The presented model provides a real-time prediction framework using neural-network-based strategies and continuum mechanics principles. A time-space integration scheme is employed to discretize the continuous dynamics and decouple the dynamic equations for translation and rotation for each node of a soft continuum manipulator. Then the resulting architecture is used to develop distributed prediction algorithms using recurrent neural networks. The proposed RNN-based parallel predictive scheme does not rely on computationally intensive algorithms; therefore, it is useful in real-time applications. Furthermore, simulations are shown to illustrate the approach performance on soft continuum elastica, and the approach is also validated through an experiment on a magnetically-actuated soft continuum manipulator. The results demonstrate that the presented model can outperform classical modeling approaches such as the Cosserat rod model while also shows possibilities for being used in practice.

## Introduction

Soft continuum manipulators are flexible and highly deformable robots composed of soft and mostly elastic materials, and can serve as possible substitutes for rigid robots. Advantages of soft manipulator robots such as their compliance, dexterity, and adaptability to complex workspaces are driving the emergent research in this field. By contrast, rigidity of traditional rigid robots limits their use in constrained and confined environments, and reduces the possibilities for safe interaction with humans. Soft continuum manipulators have found applications in many areas, such as dexterous grasping (McMahan et al., 2006; Katzschmann et al., 2015) and assistive devices (Ansari et al., 2017), and particularly in the field of minimally invasive surgeries, such as laryngeal surgery (Simaan et al., 2004), catheter-based endovascular intervention (Grady et al., 2000; Burgner et al., 2013), and cardiovascular surgery (Kesner and Howe, 2011).

Analytical modeling of soft manipulators helps evaluate their motion and determine their workspace, in order to be used for control, motion planning, and animation purposes. Soft manipulators distinguish themselves by having an infinite number of degrees of freedom in any workspace they occupy. This characterization makes modeling complicated for soft manipulators. Several approaches have been investigated thus far in the literature. Most of the approaches consider the kinematic (i.e. static or quasi-static) modeling of the manipulators such as static analysis using virtual-work model (Xu and Simaan, 2010), Cosserat rod theory (Pai, 2002; Jones et al., 2009; Mahvash and Dupont, 2011), and *α* Lie group formulation (Grazioso et al., 2019). These models do not describe full dynamics of the manipulators, and they may show performance degradation when it comes to high-frequency applications or large and complex deformations. On the other hand, dynamical modeling approaches [e.g. Wen et al. (2012), Jung et al. (2014), Hyatt et al. (2019), Sadati et al. (2019), Till et al. (2019), Tariverdi et al. (2020)], contain dynamics of the manipulators and also take into account time-varying responses of manipulators, including high-frequency modes. However, the dynamic models mostly rely on traditional methods, such as finite elements and finite differences (i.e., quantitative and numerical methods), making the algorithms computationally expensive for real-time applications. In other words, to obtain sufficiently accurate solutions, methods need to deal with fine meshes, which increase memory use and computation time. Another limitation is that their solutions are discrete or not sufficiently differentiable. It is worth noting that in model-based controllers or observers, having a differentiable solution (i.e., a solution that can be evaluated continuously on the workspace) is crucial in the design process. Furthermore, when softer materials are employed for manipulator construction with more complex geometries or large deformations, modeling their behavior analytically becomes challenging. Therefore, there is a need for appropriate data-driven approaches without compromising computational bandwidths and the prediction quality.

Dynamics of soft continuum manipulators have highly nonlinear behavior and are expressed as Partial Differential Equations (PDEs). An effective approach to represent and model PDEs solutions is to use Neural Networks (NN). NN-based solutions of PDEs are infinitely differentiable by eliminating the need for interpolation. Furthermore, compared to finite elements or difference methods, solutions are represented by fewer parameters, which reduces the memory use. There are studies that use machine learning algorithms to find a solution for special types of PDEs such as (Lagaris et al., 1998; Lee and Kang, 1990; Weinan et al., 2017; Raissi et al., 2019). However, to the authors’ best knowledge, there is no study that investigates possible NN-based solutions for partial differential equations that describe the full dynamics of continuum manipulators. In this work, inspired by a time-space integration scheme and by using the Lie group variational integration method (Demoures et al., 2015), the dynamic equations for translation and rotation for each node of a soft continuum manipulator are decoupled, providing an appropriate structure aimed at developing a real-time modeling algorithm. Afterward, Recurrent Neural Networks (RNNs)-based models are employed to approximate the high-dimensional discretized equations. Additionally, external torques and forces (e.g., control inputs, friction, and gravity) are incorporated into the model in a real-time manner for control applications.

The ability of RNNs to learn and approximate large classes of nonlinear functions over sequences of inputs accurately makes them prime candidates for use in dynamic modeling of complex nonlinear systems. RNNs with Long Short-Term Memory (LSTM) layers process sequences by iterating through the sequence elements. Using an internal feedback, the network is capable of preserving long-term dependencies. Essentially, LSTM layers prevent older information from gradually vanishing. These networks also have been used for several applications in soft robotics. To name a few, Thuruthel in Thuruthel et al. (2019) proposes a model-free, real-time sensing method for soft robots perception. The authors in Thuruthel et al. (2017) uses RNNs to model and control soft robotic manipulators. Also, force and motion estimation using RNNs has been investigated in Marban et al. (2019) and Turan et al. (2018), respectively.

This paper aims to develop a real-time dynamic model for analyzing the dynamics of soft manipulators. Investigation of previous work on the modeling of the continuum manipulators suggests that existing literature focuses primarily on static or quasi-static approaches, or does not provide a real-time model. The contribution of this article is to present a scalable, parallel and real-time modeling algorithm for soft manipulators dynamics. The contributions of this paper are as follows.

• Existing approaches primarily deal with kinematic modeling methods. Nevertheless, in this study, real-time prediction of soft manipulators full spatial dynamics is considered in the proposed RNN-based algorithm by proposing multiple light-weight RNN-based models.
• In traditional modeling approaches, there are no systematic methods to obtain knowledge about dissipation forces, in particular friction, in the modeling procedure. The presented algorithm intrinsically takes the dissipation forces into account and incorporates their effects into the model.
• Through an experiment, results of the proposed RNN-based model and Cosserat rod theory method are compared, revealing the practical effectiveness of the proposed methodology.

The remainder of this paper is organized as follows: the problem statement is given in Section 2. Section 3 describes the proposed RNN-based algorithm in details. In Section 4 and Section 5, different simulations and experimental validation are presented to demonstrate the efficacy of the proposed RNN-based method, in terms of the model performances and accurately predicting poses of manipulators. Finally, the main conclusions are stated in Section 7.

## 2 Problem Statement

Consider a continuum manipulator with large deflections described by dynamic equations of motion [as presented in Tariverdi et al. (2020) and Demoures et al. (2015)] in the PDEs form as

$$\begin{array}{l} H\omega_{t}+\omega \times H\omega +n\times \Lambda^{-1}\phi_{x}-\Lambda^{-1}\Lambda_{x}\times m-m_{x}=\Lambda^{-1}\tau \\ M\phi_{tt}-\Lambda \left(\Lambda^{-1}\Lambda_{x}\times n\right)-\Lambda n_{x}+f^{c}=f \end{array}$$ (1)

where M=ρ×A (*ρ* and *A* are the manipulator constant mass density and its cross-section area), $\omega \in {\mathbb{R}}^{3}$ is the manipulator’s angular velocity, $H\in {\mathbb{R}}^{3\times 3}$ is the manipulator’s inertia matrix, $\phi \in {\mathbb{R}}^{3}$ is the position of the manipulator’s line of centroids in its workspace, Λ∈SO(3) denotes the orientation of moving cross-sections at point ϕ. Also, $n\in {\mathbb{R}}^{3}$ and $m\in {\mathbb{R}}^{3}$ are the stresses and momenta along the manipulator, $f^{c}\in {\mathbb{R}}^{3}$ represents conservative forces (e.g. gravity). Furthermore, ${\left(\cdot \right)}_{x}$, ${\left(\cdot \right)}_{t}$, and ${\left(\cdot \right)}_{tt}$ denote partial derivatives with respect to position, time, and the second partial derivative with respect to time, respectively. Finally, $f\in {\mathbb{R}}^{3}$ and $\tau \in {\mathbb{R}}^{3}$ are non-conservative forces and torques (e.g., frictions and control inputs)^1^.

Although high fidelity models given in the references can describe continuum manipulators dynamics efficiently, they suffer from limitations that are discussed in Section 1. Inspired by the structure and formulation of the dynamics based on the Lie group variational integration scheme, the aim is to propose distributed deep recurrent neural networks to capture and simulate soft manipulators dynamics in real-time to be able control them more accurately than existing models.

## 3 Proposed RNN-Based Model

This section is devoted to develop a model based on the time series prediction using RNNs. To solve PDEs numerically using NNs, one approach is to utilize discrete solutions of finite element or difference methods to train an NN. A Lie group variational time integration model is employed to discretize the continuous dynamics of a soft manipulator^2^. The whole manipulator is discretized into an arbitrary number of nodes where the position and orientation equations of each node are decoupled. In our study, we discretize the manipulator with equidistant nodes, but this can be changed depending on the application.

**FIGURE 1:**
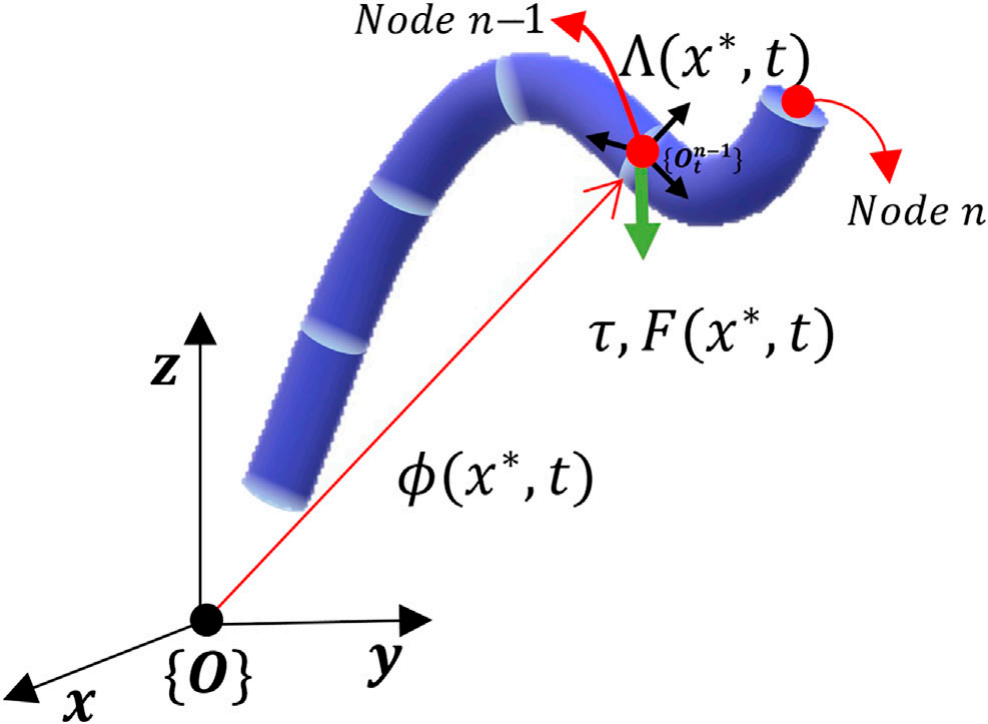
A soft manipulator at time *t* with discretization nodes *n* and n−1 are shown. $\phi \left(x^{\text{*}},t\right)$ and $\Lambda \left(x^{*},t\right)$ denote the position and the orientation of cross-section of Node n−1, respectively. In addition, the force $F\left(x^{\text{*}},t\right)$, torque $\tau \left(x^{\text{*}},t\right)$, and the conservative force $f^{c}$ (e.g., gravity) are applied to Node n−1 at the position $\phi \left(x^{\text{*}},t\right)$.

Figure 1 demonstrates a soft continuum manipulator at time *t* where $x^{\text{*}}$ is the undeformed length of Node n−1. The force $F\left(x^{*},t\right)$, torque $\tau \left(x^{*},t\right)$ are applied to Node n−1 at the position $\phi \left(x^{*},t\right)$. Also, $\Lambda \left(x^{*},t\right)$ is the orientation matrix from the frame {O} to the frame $\left\{O_{t}^{n-1}\right\}$ attached to the cross-section of Node n−1.

**FIGURE 2:**
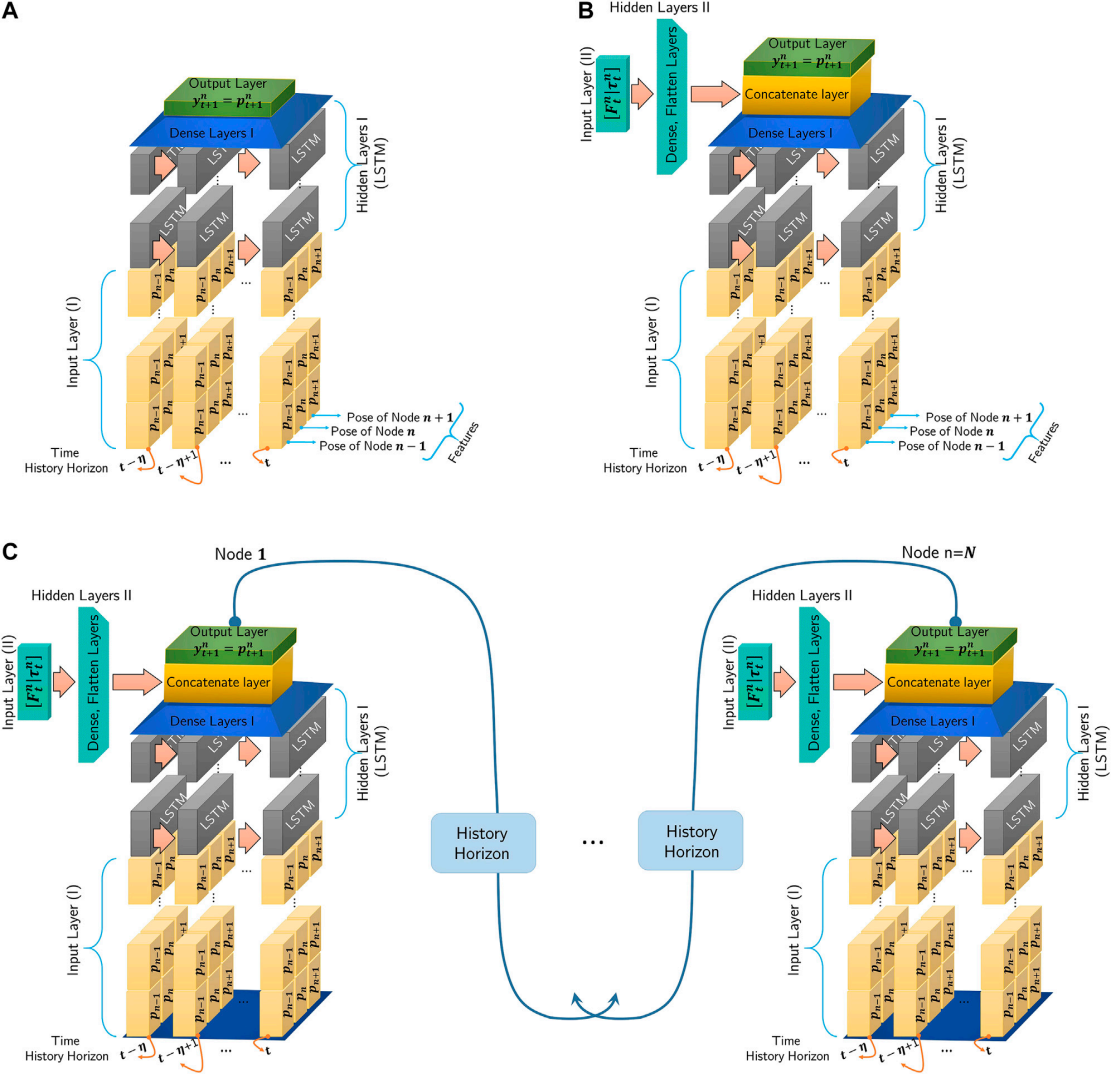
Recurrent Neural Network-based model, length of the time history horizon is determined by *η* and features composed of adjacent nodes pose: **(A)**: Poses of Nodes n−1, *n*, and n+1 are the input layer and no forces or torques are applied to the node. **(B)**: The first input layer is composed of poses $p_{n-1}$, $p_{n}$, and $p_{n+1}$ at time history horizon [t−η,t−η+1,…,t] and the second input layer includes forces and torques ${\left[F_{t}^{n},\tau_{t}^{n}\right]}^{T}$ which are incorporated into the model through the Hidden Layers II (dense and flatten layers). **(C)**: Proposed RNN-based models of the continuum manipulator with *N* nodes including Input, Hidden, and Output Layers. A history of each node output is used as an input for adjacent nodes. Nodes poses (Input Layer I) and forces and torques (Input Layer II) through the Hidden layers I and II are proceed and concatenated together.

The discrete equations suggest an appropriate structure for the RNNs-based model. Given time-sequence inputs (as a first input layer), i.e., poses (positions and orientations) of nodes, and also forces and torques (as a second input layer) applied to each node, the RNN-based model of Node *n* is depicted in Figure 2A,B. For Node *n*, the first input layer is a time-sequence series of poses $p_{n-1}$, $p_{n}$, and $p_{n+1}$ (i.e., poses of Node *n* and its adjacent nodes n−1 and n+1) and the second input layer includes forces and torques of node *n* at time *t*, i.e., ${\left[F_{t}^{n},\tau_{t}^{n}\right]}^{T}$ which are incorporated into the model through dense layers.

The network takes specific size vectors as inputs, which are called input layers. The inputs are transformed through a series of hidden layers (LSTM, dense, or fully connected layers) to produce an output. The output vectors are called an output layer. Dense or fully connected layers perform linear operations (i.e., multiplication and summation) on their inputs. Furthermore, LSTM layers consist of LSTM units, which can process sequences of data of any length, for example, poses of nodes. An LSTM unit controls contributions of each element of the input layer in the output and keeps track of the dependencies between the elements (Hochreiter and Schmidhuber, 1997).

For the training process, data-sets contain time-sequence inputs and forces and torques applied to each node. Also, for each node, the poses of the node and its neighbors are considered features, as shown in Figure 2A,B. The first and second input layers proceed through LSTM layers and dense layers as hidden layers, respectively. Finally, output layers have resulted from fully connected layers.

By augmenting the given models for all nodes (see Figure 2B) as a series, the proposed RNN-based models of the whole continuum manipulator with *N* nodes with non-conservative forces and torques are depicted in Figure 2C. Output of every node is updated at each time step by using a history (at least two previous time steps) of neighboring node outputs. Therefore, the proposed architecture suggests a suitable framework to construct a parallel modeling algorithm.

## 4 Simulations Results

In this section, we consider different examples and evaluate the performance of the proposed RNN-based model in Figure 2C. It is worth mentioning that data-sets play a crucial role in efficiency and accuracy in machine learning-based algorithms. The data acquisition process from a robot in real-world environments is both time and cost-consuming (implementation of multiple sensors, data filtering, and fusion, etc.). As an alternative approach, the required data can be acquired through simulations of high fidelity models. The obtained data can thus be transferred to train the algorithms to be implemented in real-world scenarios. In this section and for the presented examples, required data for the training of the proposed RNN-based model are acquired through simulations of the algorithm presented in Tariverdi et al. (2020), Sec. 2). For clarity, this model is henceforth referred to as the analytical dynamic model. In addition, since thin rods are considered in the examples, orientations of cross-sections are not of any concern. Also, it should be noted that orientations, except the twisting angle, can be reconstructed from manipulators’ configuration. Therefore, to obtain a computationally light model, the focus of attention is only on the prediction of positions.

### 4.1 First Simulation: An Ellipse Without External Wrenches

As a first case, a cylindrical rod is bent into a circle and its ends are attached to one another. The rod is then deformed into an elliptical shape and released. Due to potential energies in the ellipse, it starts to move without any external disturbances. The goal is to model the behavior of the ellipse resultant from its internal elastic energy.

**FIGURE 3:**
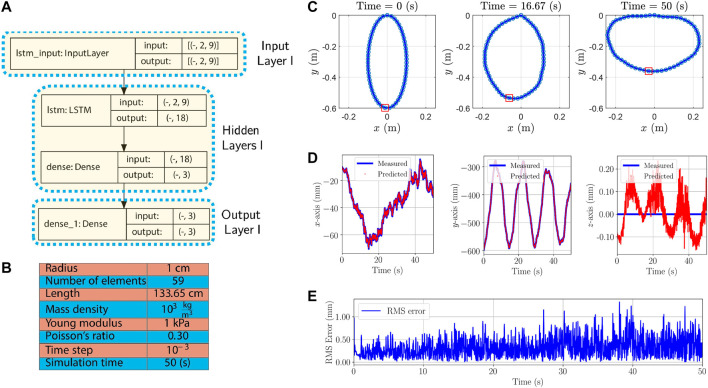
First simulation example: **(A)**: The model architecture used for the first simulation example. The first stage is the Input layer, the intermediate stages are the Hidden layers, and the last stage is the Output layer. The first dimension of inputs and outputs in each layer are unspecified and can vary with the size of batches. **(B)**: Rod properties and simulation parameters used in the first example. **(C)**: Initial and time-evolved configurations. Positions of node specified by a red rectangle are measured and predicted. **(D)**: Measured position calculated from analytical dynamic model and predicted by the proposed RNN-based model in the x, y, and z-axes are given. **(E)**: RMS error considering all the axes.

The ellipse is formed in the xy-plane with the width 0.2 m and height 0.6 m. As boundary conditions, the first and last nodes are fixed to the origin and their orientations are set to $R_{y}\left(18.07{}^{\circ}\right)$ and $R_{y}\left(341.92{}^{\circ}\right)$, respectively, where $R_{y}\left(\theta \right)$ denotes a rotation matrix describing a rotation around the *y*-axis by *θ* degrees. The rod properties, simulation parameters, and the structure of the proposed RNN-based model are given in Figure 3A,B. Furthermore, the initial and a few time-evolved configurations are shown in Figure 3C. As seen, the ellipse oscillates back and forth due to its internal elastic energy. Since orientations except the twisting angle can be reconstructed from the configuration of the manipulator, to have a light model and for brevity, positions of the node located at (−0.01,−0.59)—Node 30th—are predicted. The chosen node is the furthest from the origin and would, compared to other nodes, most likely have the largest errors.

50,001 position samples are generated from the analytical model for each node. We augment 1-by-3 position vectors of Node 30th and its adjacent nodes (Nodes 29th and 31st) at each time step. Therefore, the augmentation results in a 1-by-9 vector. Furthermore, the size of history horizons is chosen to be 2. In other words, η=1 in Figure 2A,B. Finally, augmented 2-by-9 tensors are obtained for each time step. The prepared data-set is called Data-set I and 60 percent of it is used for training process. The architecture in Figure 3A shows the input layer consists of tensors of size 2 × 9. The first dimension of all layers are reserved for batch sizes and for the training, the batch size 1 was chosen. In the architecture of the model in Figure 3A, the Input, Hidden and Output Layers I together with the number of nodes and type of layers are demonstrated according to Figure 2A.

First, we evaluate the model by using unseen data samples in Data-set I and the results are shown in Figure 3D. The maximum and mean absolute error are (1.57 mm, 0.27 mm), (2.27 mm, 0.46 mm), and (0.23 mm, 0.06 mm), or in other words, the percentage of the maximum errors with respect to the length of the manipulator are 0.11, 0.17, and 0.02 in the x, y, and z-axes, respectively. It is worth mentioning that it is prior knowledge that the manipulator does not have any motion in the z-axis and therefore, components of the z-axis in position vectors can be ignored. Furthermore, the evaluation Root-Mean-Square (RMS) errors of the considered node in all axes at every time step is calculated by

$$RMSE\left(t\right)=\sqrt{\frac{1}{3}\left({\left(x_{p}\left(t\right)-x_{m}\left(t\right)\right)}^{2}+{\left(y_{p}\left(t\right)-y_{m}\left(t\right)\right)}^{2}+{\left(z_{p}\left(t\right)-z_{m}\left(t\right)\right)}^{2}\right)}$$ (2)

where predicted positions ${\left[x_{p}\left(t\right),y_{p}\left(t\right),z_{p}\left(t\right)\right]}^{T}$ obtained from the proposed RNN-based model and measurement positions ${\left[x_{m}\left(t\right),y_{m}\left(t\right),z_{m}\left(t\right)\right]}^{T}$ in Data-set I and the results are shown in Figure 3E.

To demonstrate that the model can be extended to different boundary and initial conditions, the cylindrical rod is employed to form a horizontal ellipse with the width 0.6 m and height 0.2 m. The rod properties and the simulation parameters given in Figure 3B are used. As boundary conditions, the first and last nodes are attached to the origin and their orientations are set to the identity. The manipulator with the new boundary and initial conditions is only used for the evaluation of the trained model by predicting positions of the node located at (−0.01,−0.19). Based on the prediction, the maximum and mean absolute errors are (26.33 mm, 3.37 mm), (21.71 mm, 3.70 mm), and (4.92 mm, 4.22 mm) in the x, y, and z-axes, respectively. Furthermore, the maximum/worst-case errors with respect to the length of the manipulator are 1.97%, 1.62%, and 0.37% in each axis, respectively.

Let us assume that the analytical dynamic model is implemented in a parallel scheme, i.e., each node of 59 nodes is handled with a CPU core or different hardware such that there is no latency in communications. Then, the dynamics of each node can be solved in $1.62\times 10^{-4}$ s on average. In addition, to preserve the convergence of the solver of the analytical dynamic model, the maximum constant time step for this simulation is $10^{-3}$ s. A minimum criterion to have a real-time performance is that the time required to solve each node dynamics must be less than the constant step simulation. To be more specific, to have a real-time model, the CPU time, i.e., the amount of time spent in a user code must be less than Wall-clock time that measures the time elapsed to run a user code. According to this minimal criterion, as long as the computation-time for simulation of a method/model is less than a user-defined time for the simulation, the model is called a real-time model. It can be shown that in this example and based on the given assumption, the maximum bandwidth for a real-time performance is 3.93 Hz on average (calculation is done on a 16 GB, 1.99 GHz Intel i7 machine running windows 10). It should be pointed out that we use the same machine for calculations in this paper. It will be discussed that even achieving this bandwidth limit is not feasible. On the other hand, for the proposed RNN-based model, the bandwidth of a real-time performance is 65.70 Hz, which can be further improved by optimizing the number of layers and trainable parameters.

It is worth mentioning that the considered assumption is very strict, which cannot be satisfied in reality. First of all, conventional algorithms need a relatively high number of nodes to have numerical stability and an acceptable convergence rate. Furthermore, due to limitations in computation resources, more than one node will be assigned to each core of CPU, and there is always latency in communications between threads in parallel programmings. Therefore, reaching the mentioned bandwidth through the analytical dynamic model is infeasible. However, the real-time performance of the proposed RNN-based model can be applicable in closed-loop control applications.

### 4.2 Second Simulation: A Cylindrical Rod With External Wrenches

**FIGURE 4:**
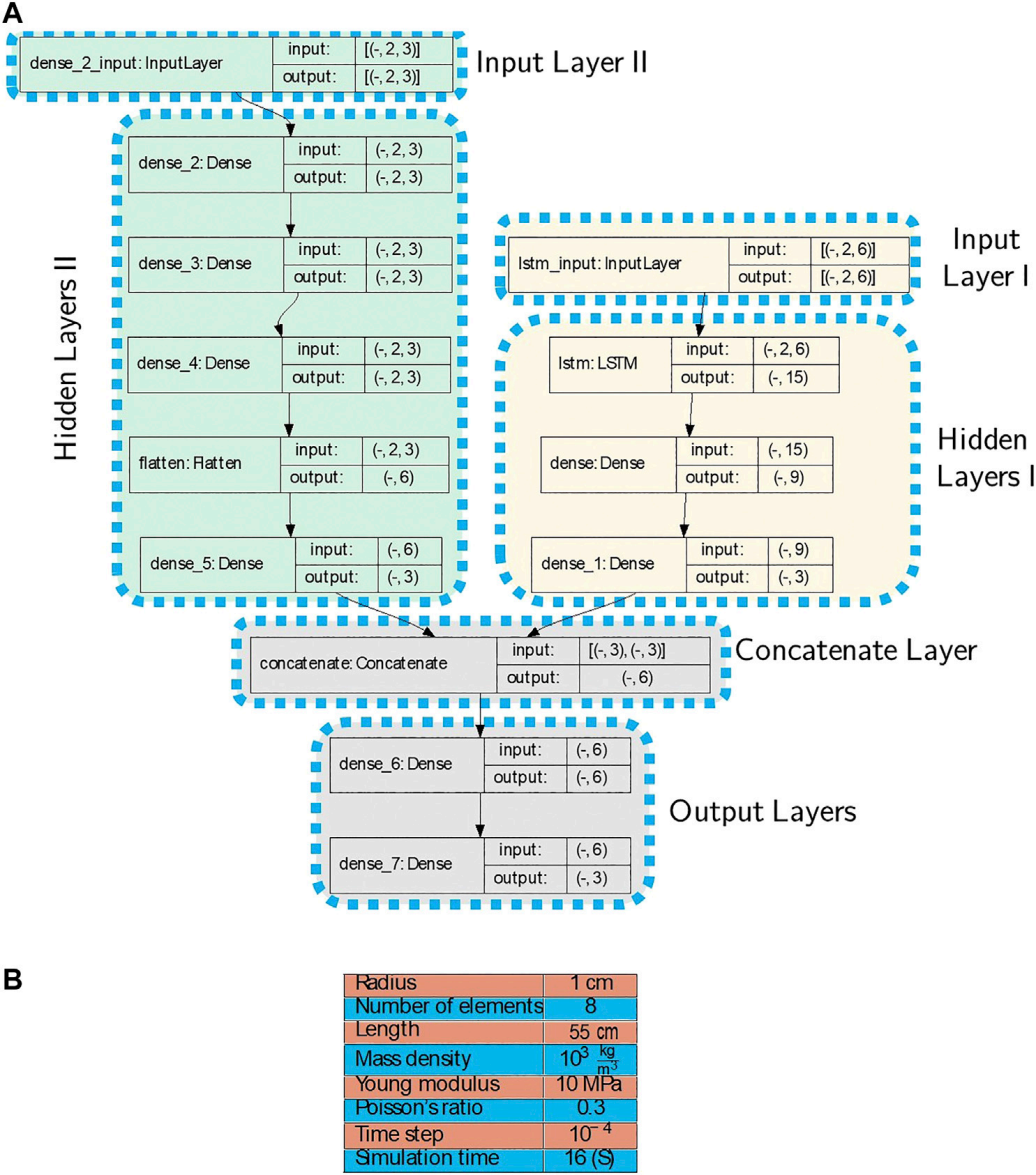
**(A)**: The model architecture used for the second simulation example. There are two Input layers, the first one is the poses of the node and the second input is the applied forces on the node. The first dimension of inputs and outputs in each layer are unspecified and can vary with the size of batches. **(B)**: Rod properties and simulation parameters used in the second example.

**FIGURE 5:**
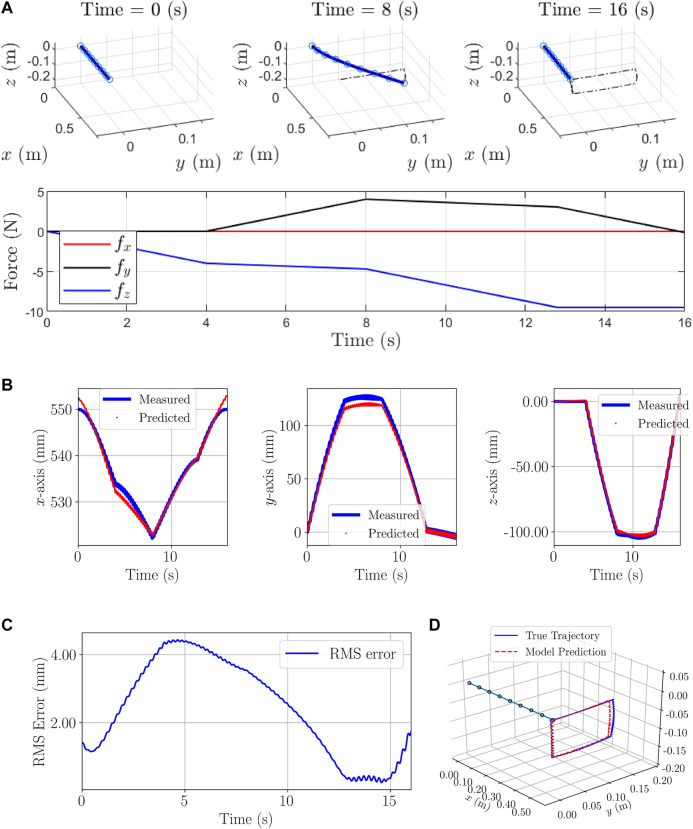
Second simulation example: **(A)**: Initial, Time-evolved configurations and forces on the last node. **(B)**: Tip positions: calculated from the analytical dynamic model and predicted by the proposed RNN-based model. **(C)** RMS error considering all the axes. **(D)** Predicted and measured trajectories.

In the second example, we simulate a rod with a circular cross section, which is actuated by external forces such that its tip tracks a square in space. In this example, the goal is to model the behavior of the rod which results from applied external forces on its end-effector. For boundary conditions, the first node is fixed to the origin and its orientation is set to the identity for all time steps. The rod properties and simulation parameters, and the structure of the proposed model are given in Figure 4. The trajectory of the end-effector and the applied forces onto it are shown in Figure 5A.

160,001 position and force samples are generated from the analytical model for each node. We augment 1-by-3 position vectors of the last node (end-effector) and its adjacent node at each time step. Therefore, the augmentation results in a 1-by-6 vector. Furthermore, the size of history horizons is chosen to be 2 (η=1). Finally, augmented 2-by-6 tensors are obtained for each time steps which are fed to the model as the Input Layer I. The same preparation process are applied for the force data samples which are used as the Input Layer II. The prepared data-set is called Data-set II and 60% of it is used for training process. The architecture in Figure 4A shows the Input layers I and II consist of tensors of size (Batch Size×2×6) and (Batch Size×2×3), respectively. The first dimension of all layers are reserved for batch sizes and for the training, the batch size 1 was chosen. In the architecture of the model in Figure 4A, the Input, Hidden and Output Layers I and II together with the number of nodes and type of layers are demonstrated according to Figure 2B.

First, unseen data samples in Data-set II are employed to evaluate the model, and tip positions are calculated and the results are shown in Figure 5B. The maximum and mean absolute error are (3.58 mm, 1.70 mm), (1.80 mm, 0.69 mm), and (2.73 mm, 1.41 mm), or in other words, the maximum errors with respect to the length of the manipulator are 0.71%, 0.36%, and 0.54% in x, y, and z-axes, respectively. The RMS errors of the end-effector through Eq. 2 are shown in Figure 5C.

**FIGURE 6:**
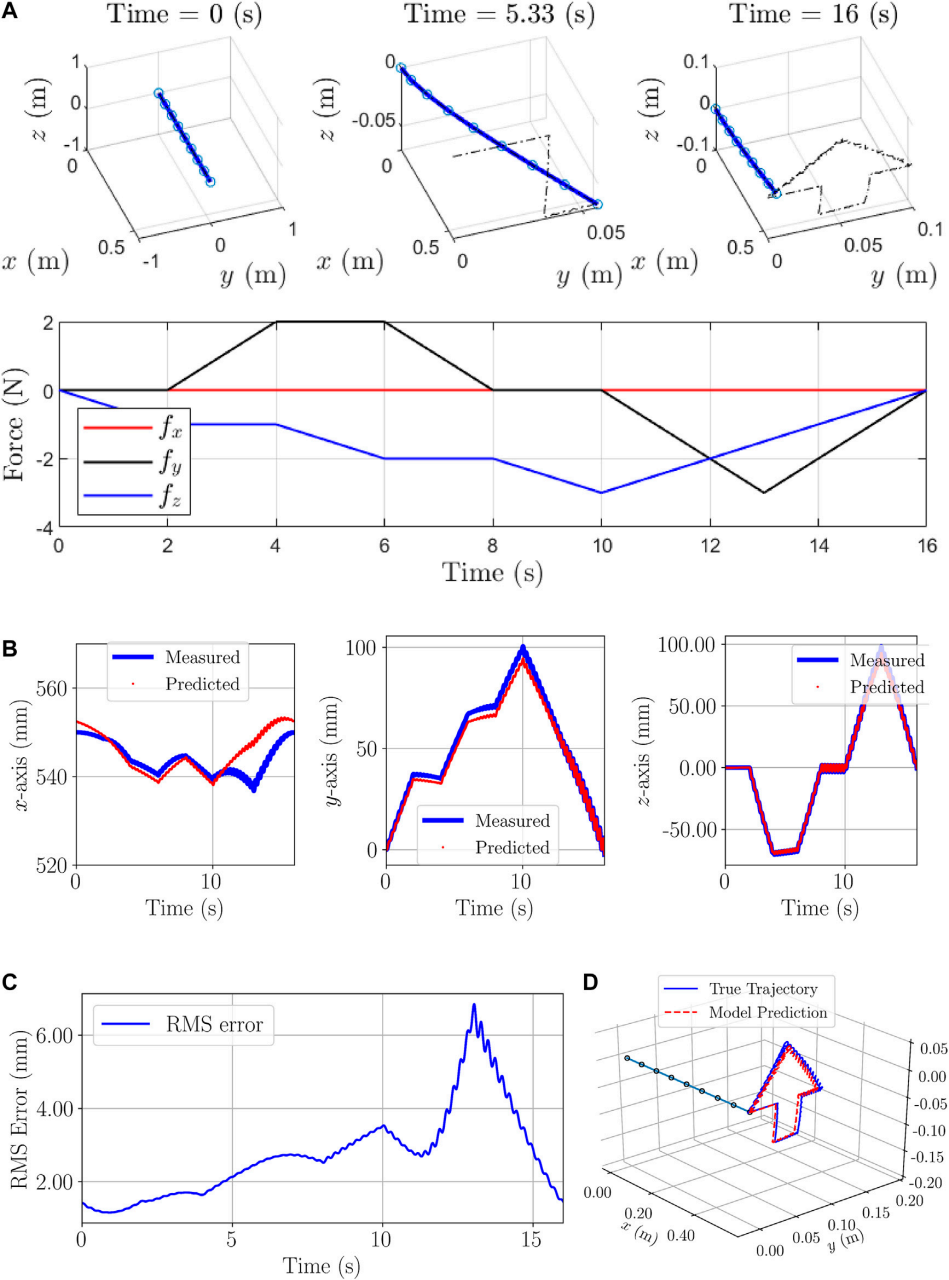
Evaluation Example of Second simulation: **(A)**: Initial, Time-evolved configurations and forces on the end-effector **(B)**: Tip positions: calculated from the analytical dynamic model and predicted by the proposed RNN-based model. **(C)** RMS error considering all the axes **(D)** Predicted and measured trajectories.

To evaluate the generalizability of the trained model, different profiles of forces are applied to the model aiming at obtaining different position trajectories for the end-effector as depicted in Figure 6A,D. To fulfill the goal of the second example, the new forces are only used for the evaluation of the trained model by predicting the positions of the end-effector. Results of the prediction are plotted in Figure 6B and are as follows: The maximum and mean absolute errors are (10.49 mm, 2.61 mm), (5.54 mm, 1.05 mm), and (5.97 mm, 2.83 mm), furthermore, the percentage of the maximum/worst-case errors with respect to the length of the manipulator are 1.90, 1, and 1.08 in the x, y, and z-axes, respectively. The RMS errors of the end-effector through Eq. 2 are shown in Figure 6C.

In this example, the maximum constant time step for this simulation is $10^{-4}$ s, to have a convergent numerical solver for the analytical dynamic model. In addition, on average, the time $1.89\times 10^{-4}$ s is required for solving the dynamics of each node. In other words, the analytical dynamic model can not achieve any real-time performance for this example. However, the proposed model achieves a real-time performance of the bandwidth of 60.30 Hz on average.

### 4.3 Third Simulation: A Cylindrical Rod With and Without External Wrenches

**FIGURE 7:**
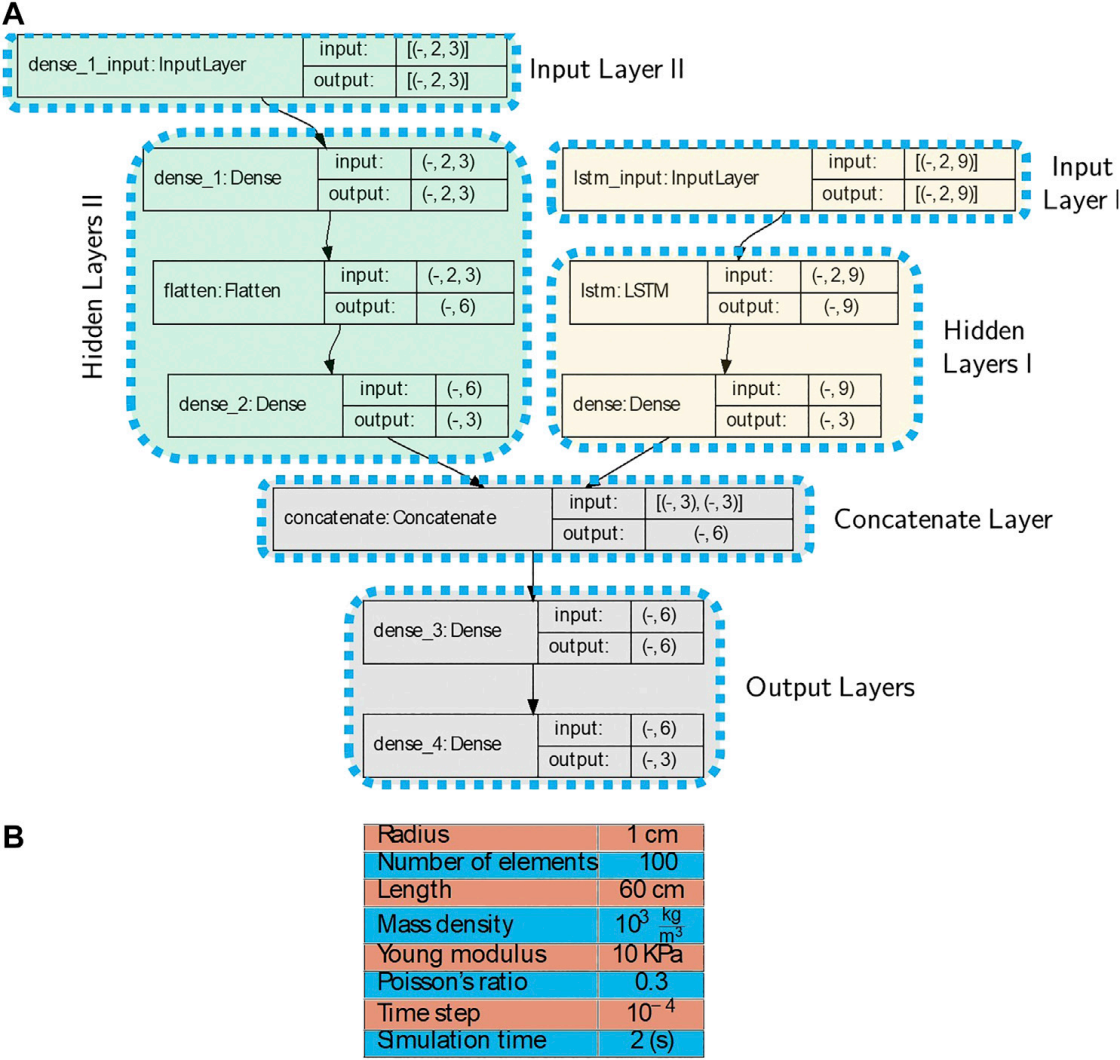
**(A)**: The model architecture used for the third simulation example. There are two Input layers, the first one is the poses of the node and the second input is the applied forces on the node. The first dimension of inputs and outputs in each layer are unspecified, and can vary with the size of batches **(B)**: Rod properties and simulation parameters used in the third example.

**FIGURE 8:**
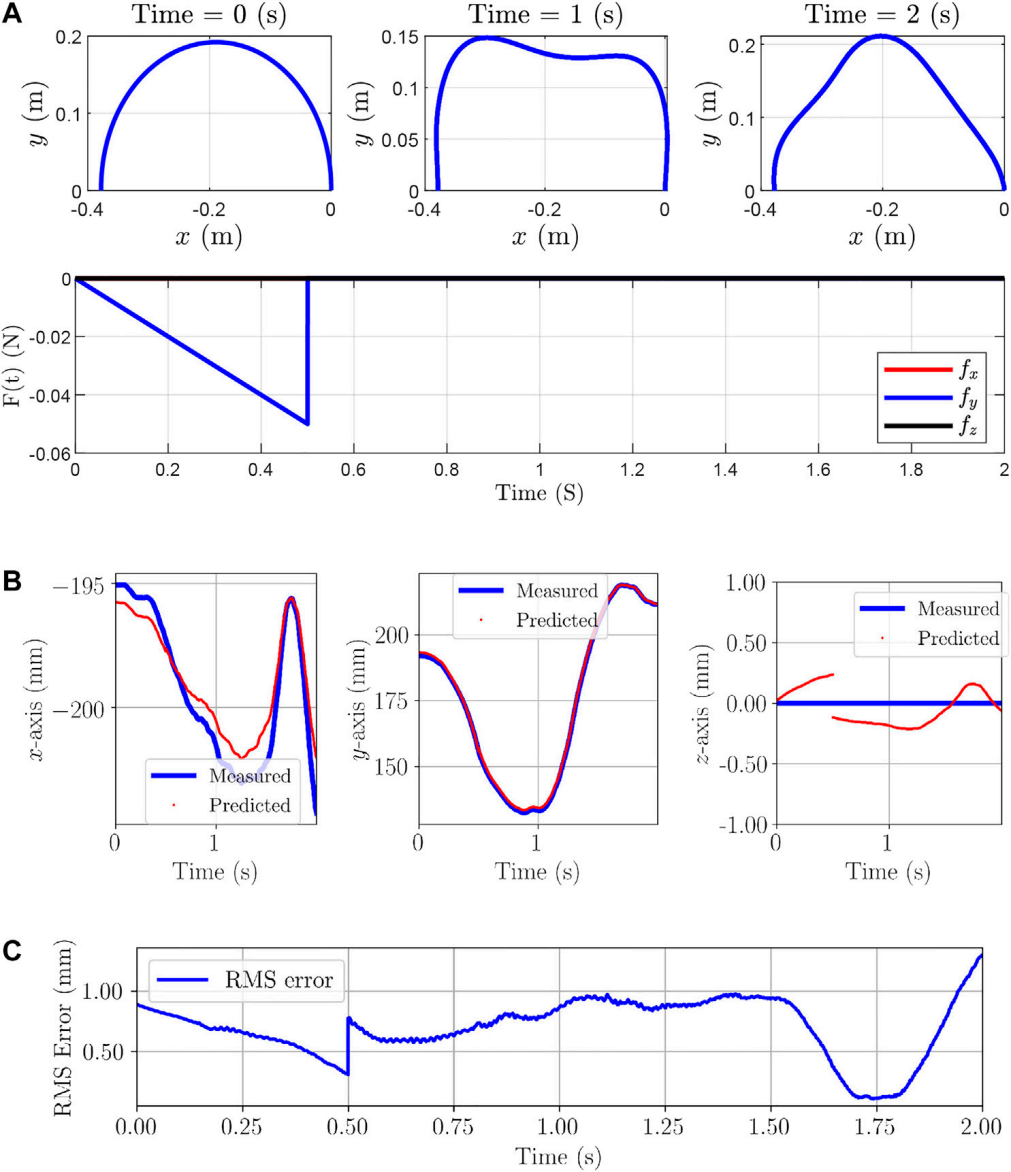
Third simulation example: **(A)**: Initial, Time-evolved configurations and forces applied on the rod. Positions of the middle node, where the force is applied, are measured and predicted. **(B)**: Measured position calculated from analytical dynamic model and predicted by the proposed RNN-based model in the x, y, and z-axes are given. **(C)** RMS error considering all the axes.

In the last example, we form a semi-circular shape with a cylindrical rod. A force is applied to the middle node—Node 51th—in the -*y*-axis direction for 0.5 s and then the force is removed. Furthermore, the boundary conditions are as follows: the first and last nodes are fixed to the origin and their orientations are set to the identity and $R_{z}\left(181.81{}^{\circ}\right)$, respectively, where $R_{z}\left(\theta \right)$ describes rotation around the *z*-axis by *θ* degrees. In this example, the idea is to model the behavior of the rod resulted from applied external forces and internal elastic energy. The structure of the proposed model is given in Figure 7A and the rod properties and simulation parameters are given in Figure 7B. The initial and a few time-evolved configurations together with the applied forces are given in Figure 8A.

20,001 position and force samples are generated from the analytical model for each node. We augment 1-by-3 position vectors of Node 15th and its adjacent nodes at each time step. Therefore, the augmentation results in a 1-by-9 vector. Furthermore, the size of history horizons is chosen to be 2 (η=1). Finally, augmented 2-by-9 tensors are obtained for each time steps which are fed to the model as the Input Layer I. The same preparation process are applied for the force data samples which are used as the Input Layer II. The prepared data-set is called Data-set III and 60% of the data is used for training process. The architecture in Figure 7A shows the Input layers I and II consist of tensors of size (Batch Size×2×9) and (Batch Size×2×3), respectively. The first dimension of all layers are reserved for batch sizes and for the training, the batch size 1 was chosen. In the architecture of the model in Figure 7A, the Input, Hidden and Output Layers I and II together with the number of nodes and type of layers are demonstrated according to Figure 2B.

The positions of Node 51th are predicted using seen and unseen data samples in Data-set III and the results are shown in Figure 8B,C. The maximum and mean absolute error are (1.36 mm, 0.78 mm), (0.23 mm, 0.13 mm), and (2.22 mm, 0.81 mm). Furthermore, the maximum/worst-case errors with respect to the length of the manipulator are 0.22%, 0.04%, and 0.37% in the x, y, and z-axes, respectively.

For the evaluation of the trained model and to fulfill the goal of this example, force vector ${\left[0,0,-100\times \cos\left(\pi t\right)\right]}^{T}\;\text{mN}$ mN is applied to Node 51th for t∈[1,2] s. Results of the prediction are as follows: The maximum and mean absolute errors are (3.73 mm, 1.25 mm), (2 mm, 0.2 mm), and (8.1 mm, 1.2 mm), furthermore, the maximum/worst-case errors with respect to the length of the manipulator are 0.62%, 0.33%, and 1.35% in the x, y, and z-axes, respectively.

In this example, the maximum constant time step for this simulation is $10^{-4}\;\text{s}$ using the analytical model. In other words, the analytical model does not show a real-time performance since, on average, the time $2.22\times 10^{-4}\;\text{s}$ is required for solving the dynamics of each node. On the other hand, the proposed model can achieve a real-time performance of the bandwidth 58.13 Hz on average.

## 5 Experimental Results

This section is devoted to the experimental validation of the presented model. To that end, we fabricated a soft manipulator on which magnetic fields are used to produce necessary forces and torques. Compared to the simulations in which positions are predicted, time-sequence input is composed of orientations of nodes in the experiment. Furthermore, to show the performance of the algorithm, results from the presented method and a Cosserat rod-based theoretical model are compared to show the efficiency of the proposed RNN-based model. The Cosserat rod model of the soft manipulator is detailed in Appendix.

### 5.1 Soft Continuum Manipulator

**FIGURE 9:**
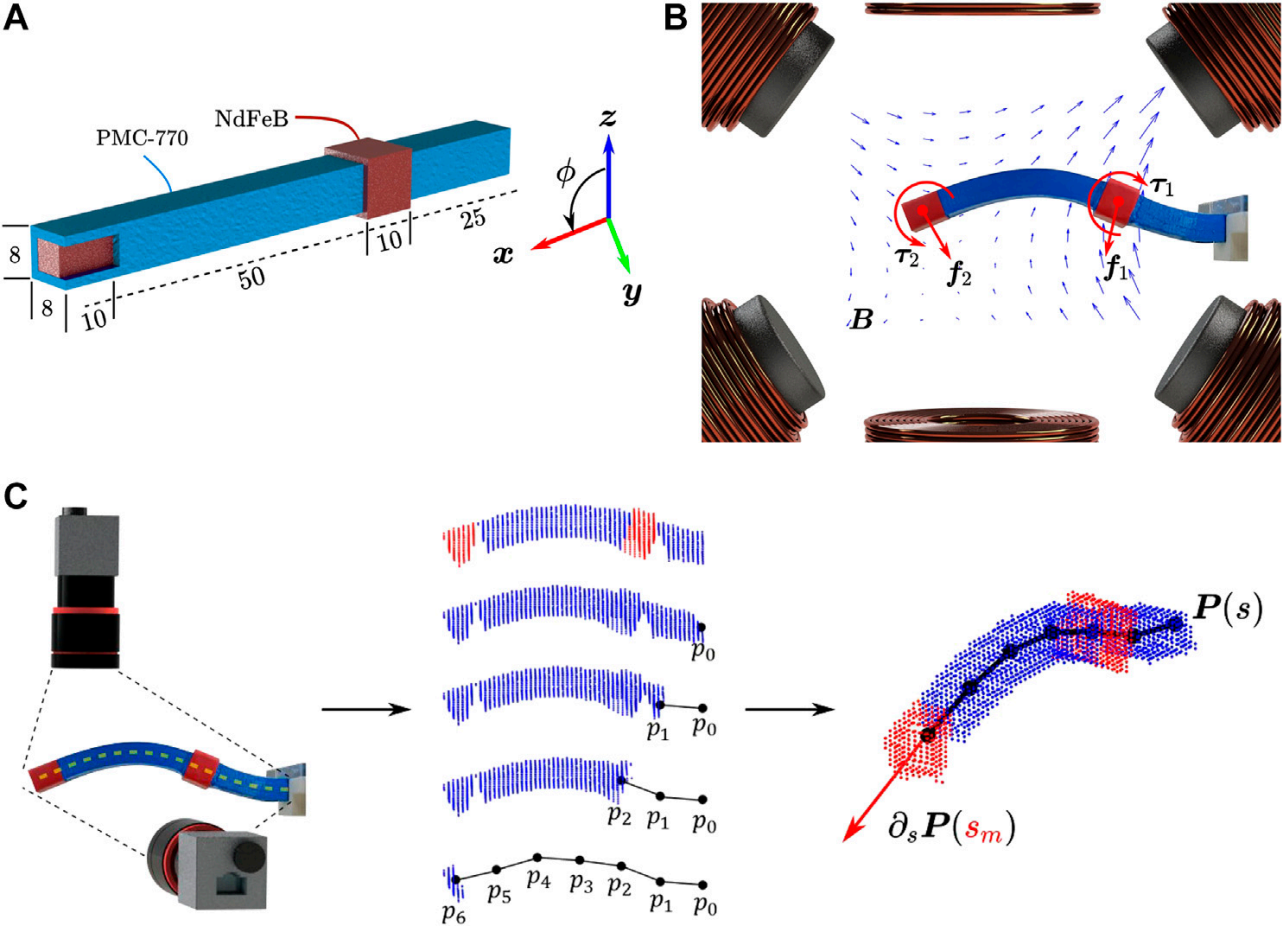
**(A)**: Polymer matrix composite 770 (PMC-770) beam continuum manipulator with embedded neodymium (NdFeb) magnets located at tip and intermediate positions. Dimensions are given in millimeter. **(B)**: Experimental setup consists of six stationary electromagnets and contains a segmented photograph of the final shape manipulator. The flexible PMC-770 and rigid NdFeb sections of the manipulator are blue and red, respectively. Six electromagnets generate a magnetic field (B) in the workspace, exerting torques and forces ($\tau_{m},f_{m}$, m=1,2) on the magnets, which deforms the continuum manipulator to its final shape at the time t=340 s. **C**: Representation of the shape reconstruction algorithm used for shape feedback. The manipulator is recorded with a stereo vision setup. The manipulator body is represented by a 3D spatial point cloud. The manipulator centerline, characterized by parameter s∈[0,L], is approximated by N+1 points $\left(\left\{p_{0},\ldots ,p_{N}\right\}\right)$. A 3D polynomial fit (P(s)) is made through the points, and the magnet orientation at an assumed constant centerline position $s_{m}$ derived from the local gradient of the polynomial fit.

A soft continuum manipulator is fabricated from a urethane rubber Polymer Matrix Composite 770 (PMC-770, Smooth-On Inc., Macungie, United States) and neodymium (NdFeB) block magnets whose dimensions are given in Figure 9A. When the manipulator is subjected to an external magnetic field, the embedded magnets experience forces and torques. This causes the flexible portions of the manipulator comprised of the PMC to undergo elastic deformation.

The PMC-770 has a density $\rho =1000\;{\text{kg/m}}^{3}$, Young modulus E=2.5 MPa, and Poisson ratio ν=0.5. The distal and proximal NdFeb magnets have grades N45 and N42, respectively. In addition, they have density ρ=7000 kg/m^3^, Young modulus E=41.4 GPa, and Poisson ratio ν=0.3. It should be pointed out that Young’s modulus and densities of the soft manipulator constituent materials were determined using a combination of supplier data and experiments until theoretical results (predicted by the Cosserat rod model) would resemble the experiment results. The magnitude of the magnetic dipoles carried by the manipulator was calculated from the magnets volume and manufacturer-supplied residual flux density.

### 5.2 Experimental Setup

The experimental setup consists of 6 stationary electromagnets surrounding a spherical workspace of 100 mm diameter Sikorski et al. (2017). Figure 9B shows the setup of the experiment. In addition, the final shape of manipulator has been segmented and is shown in the workspace. The continuum manipulator is suspended horizontally (along x) in the workspace and actuated to move in a plane, steering the magnets by manipulating the magnetic field generated by the electromagnets. Orientations are represented using the axis-angle notation. Let $k_{m}\in {\mathbb{R}}^{3}$ and $\phi_{m}\in \mathbb{R}$ denote the axis- and angle-of-rotation, respectively, where m=1,2 denotes the magnet index counting from the manipulator base. In the 2D experiment, $k_{m}=y$, and $\phi_{m}$ is defined relative to z.

Figure 9C represents the shape reconstruction of the soft manipulator through images coming from two Dalsa Genie Nano C1940 Red-Green-Blue (RGB) cameras (TeledyneDalsa, Waterloo, ON, Canada). The flexible PMC-770 and rigid NdFeb magnets were colored blue and red, respectively. The RGB cameras (horizontal and vertical) that formed a stereo vision setup recorded the workspace during experiments. First, we discretize the actuation workspace into voxels. The silhouette of the continuum manipulator is segmented as binary masks and the manipulator body represented as a 3D spatial point cloud. The manipulator centerline is approximated by N∈ℕ discrete segments. A simple iterative shape reconstruction algorithm Sikorski et al. (2019) moves through the voxels to represent the manipulator centerline with *N* discrete points $\left(\left\{p_{0},p_{1},\ldots ,p_{N}\right\}\right)$ as a function of centerline parameter s∈[0,L]. To be specific, with knowledge of the RGB-camera frames, the points are projected onto each camera image. If a point is projected onto both binary masks, the point falls within the manipulator. This process is repeated for all voxels. Subsequently a 3D polynomial fit (P(s)) is made through the points. We assume that magnetically exerted forces and torques are insufficient for the manipulator extension along the centerline, and therefore assume constant positions of the magnets along the centerline, $s_{m}\in \left(0,L\right)$. The measured magnet position is thereafter obtained from the polynomial fit $\left(p_{m}=P\left(s_{m}\right)\right)$, and its orientation from the local gradient of the polynomial fit $\left(\partial_{s}P\left(s_{m}\right)\right)$ relative to a reference z axis,

$$\begin{array}{l} \phi_{m}=\cos^{-1}\left(z\cdot {\left[\partial_{s}P\left(s_{m}\right)\right]}^{\wedge }\right)\\ k_{m}={\left[z\times \partial_{s}P\left(s_{m}\right)\right]}^{\wedge } \end{array}$$

where ${\left[\cdot \right]}^{\wedge }$ represents a normalization. Furthermore, in the experiments performed for this study, camera occlusions did not occur.

The magnetic torques and forces were computed from the magnets position $p_{m}$, magnets dipole moments $\mu_{m}\in {\mathbb{R}}^{3}$, and electromagnet currents $I_{C}\in {\mathbb{R}}^{6}$. The magnets position and orientations were obtained from the stereo vision setup. Afterwards, the orientations are used to compute magnets dipoles. To compute the magnetic field, each electromagnet is associated with a unit-current field and field gradient map ($\beta_{i}\left(p\right)\in {\mathbb{R}}^{3}$ and $\beta_{\nabla ,i}\left(p\right)\in {\mathbb{R}}^{3\times 3}$, i=1,…,6), which computes the unit-current contribution of the electromagnet to the field at field point p. We define a map $G\left(\beta_{\nabla ,i}\right):{\mathbb{R}}^{3\times 3}\to {\mathbb{R}}^{5}$ which takes the five independent gradient terms of the field (Petruska and Nelson, 2015). The field (gradient) at magnet position $p_{m}$ is then given by the superposition principle

$$\begin{array}{l} B_{m}=\left[\beta_{1}\left(p_{m}\right),\ldots ,\beta_{6}\left(p_{m}\right)\right]I_{C}\\ G\left(B_{\nabla ,m}\right)=\left[G\left(\beta_{\nabla ,1}\left(p_{m}\right)\right),\ldots ,G\left(\beta_{\nabla ,6}\left(p_{m}\right)\right)\right]I_{C}. \end{array}$$

**FIGURE 10:**
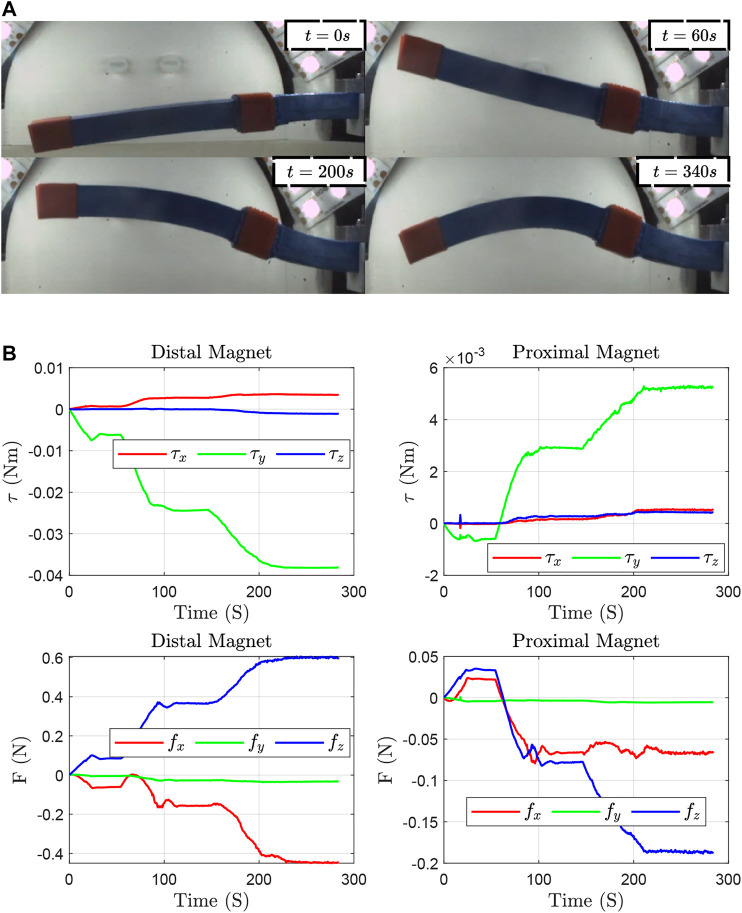
**(A)**: Initial and time-evolved configurations. **(B)**: Applied torques and forces on the distal and proximal magnets.

The torques and forces exerted on the magnets due to the field is given by

$$\begin{array}{c} \tau_{m}=\mu_{m}\times B_{m}\\ f_{m}=\nabla \left(\mu_{m}\cdot B_{m}\right) \end{array}\Rightarrow \begin{array}{c} \tau_{m}={\left[\mu_{m}\right]}_{\times }B_{m}\\ f_{m}=M\left(\mu_{m}\right)G\left(B_{\nabla ,m}\right) \end{array}$$

where $M\left(\mu_{m}\right):{\mathbb{R}}^{3}\times {\mathbb{R}}^{3\times 5}$ represents a map of the field independent spatial gradients to forces on the dipole $\mu_{m}$ (Petruska and Nelson, 2015). The applied magnetic forces and torques together with the initial and a few time-evolved configurations are shown in Figure 10.

For modeling, we consider three nodes located at the locations of the proximal and distal magnets, and the clamped end of the rod. It should be pointed out that the performance of the proposed RNN-based model, unlike conventional algorithms, is independent of the number of nodes considered for the whole manipulator. Therefore, it is sufficient to model points of interest. The idea is to independently manipulate each magnet (actuation point). However, the setup provides us with 8 degrees of freedom, meaning that positions and orientations (12 degrees of freedom) cannot be manipulated at the same time. Therefore, we carried out the experiment to achieve only orientation control.

**FIGURE 11:**
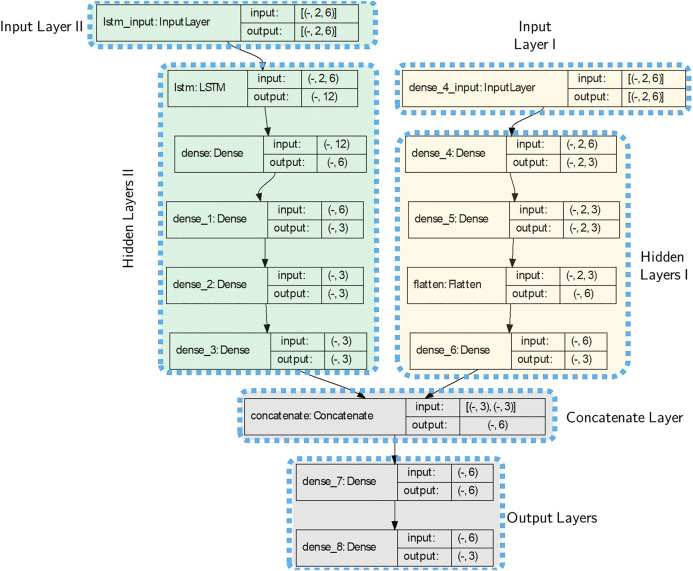
The model architecture used for the experiment. There are two Input layers, the first one is the poses of the node and the second input is the applied forces on the proximal node. The first dimension of inputs and outputs in each layer are unspecified, and can vary with the size of batches.

669 1-by-3 position samples and 1-by-6 augmented wrench samples (i.e., ${\left[\tau ,f\right]}^{T}$) for the both magnets are obtained. By choosing the size of history horizon 2 (η=1), the augmented 2-by-6 position tensors are reshaped for each time step and fed to the model as the Input Layer I. The same preparation process is applied for the force data samples which are used as the Input Layer II. The prepared data-set is called Data-set IV and 60% of the data is used for training process. We suggest the same model for both moving nodes and the architecture of the model is depicted in Figure 11 which is the same for the proximal and distal nodes. The architecture shows the Input layers I and II consist of tensors of size (Batch Size×2×6). The first dimension of all layers are reserved for batch sizes and for the training, the batch size 1 was chosen. In the architecture of the model in Figure 11, the Input, Hidden and Output Layers I and II together with the number of nodes and type of layers are demonstrated according to Figure 2B.

## 5.3 Results

**FIGURE 12:**
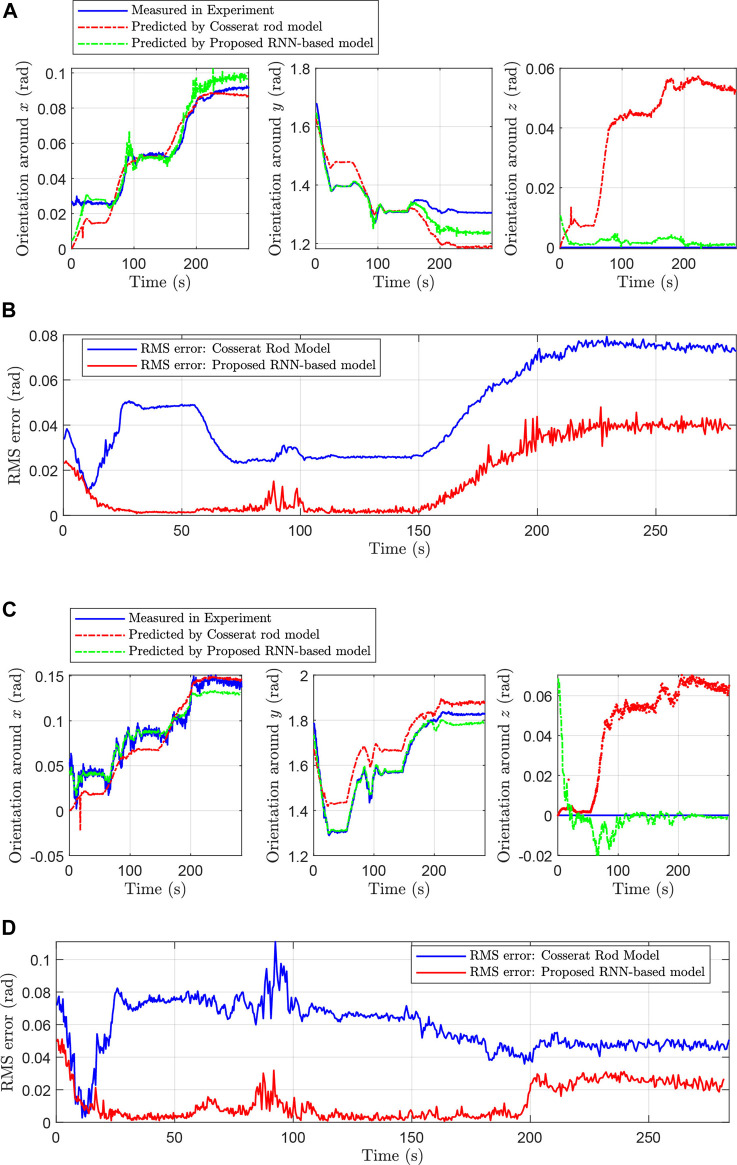
**(A)**: Measured and predicted orientations of tip/distal magnet by Cosserat rod model and proposed RNN-based model. **(B)**: RMS error for distal/tip magnet resulted from Cosserat rod model and proposed RNN-based model. **(C)**: Measured and predicted orientations of middle/proximal magnet by Cosserat rod model and proposed RNN-based model. **(D)**: RMS error for middle/proximal magnet resulted from Cosserat rod model and proposed RNN-based model.

The distal and proximal node rotations are predicted both by Cosserat rod model and the proposed model, and the results are shown in Figure 12. Also, the maximum and mean absolute errors are stated in an ordered pair in Table 1.

**TABLE 1:** The maximum and mean absolute errors around the x, y, and z-axes in ordered pairs for the distal and proximal nodes.

	*x*-axis	*y*-axis	*z*-axis
**Results for distal node**			
Proposed RNN-based model	(1.27°,0.23°)	(4.69°,1.27°)	(0.60°,0.10°)
Cosserat rod model	(3.56°,0.82°)	(10.55°,5.03°)	(4.02°,2.22°)
**Results for proximal node**			
Proposed RNN-based model	(1.13°,0.31°)	(3.30°,0.89°)	(3.93°,0.31°)
Cosserat rod model	(1.55°,0.36°)	(7.22°,3.51°)	(3.29°,1.99°)

The computation time required to find a solution of the manipulator statics from a Boundary Value Problem (BVP) with Cosserat rod theory depends on the quality of the initial solution guess, i.e., n(s) and m(s) at s=0, the tolerable error (E∈ℝ), and the number of nodes (N∈ℕ) used to discretize the manipulator.

A tolerable error describes the error between the distal internal forces and moments obtained from forward integration which are called $n_{f}^{d}$ and $m_{f}^{d}$, and distal boundary condition, i.e., $n_{b}^{d}$ and $m_{b}^{d}$. The tolerable error can be written as ${\left[\begin{array}{cc} n_{f}^{d}-n_{b}^{d}, & m_{f}^{d}-m_{b}^{d} \end{array}\right]}_{2}\leq E$.

Decreasing the tolerable error increases the solution accuracy, but potentially requires more time to solve convex optimizations for the BVP. Increasing the number of nodes is necessary to describe complex manipulator geometries, but should be chosen to minimize the required steps during forward integration.

**FIGURE 13:**
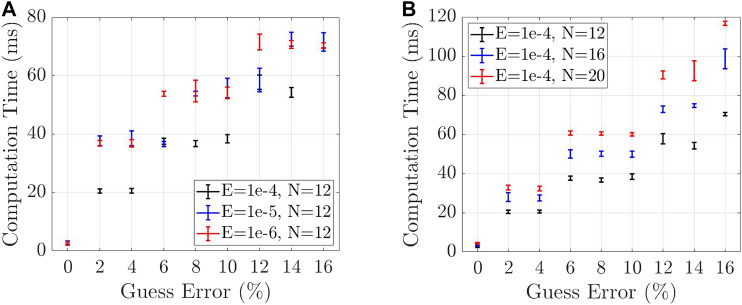
**(A)**: Computation time required for solving a solution to the BVP changes with decreasing tolerable error **(*E*)** for a constant number of nodes. **(B)**: Computation time required for solving a solution to the BVP changes with an increasing number of nodes (*N*) and increasing percentage errors from a valid solution, for a constant tolerable error.

To visualize how the required computation time changes with the number of nodes and the tolerable error, multiple simulations were performed by assigning known torques $\tau_{m}$ and forces $f_{m}$ for m=1,2, to the manipulator, and finding a valid solution from solving the BVP. Changes to tolerable errors (*E*) and number of nodes (*N*) were made manually. For example, an error of 2% in the initial solution guess was obtained by multiplying the valid solution with 0.98. After each change the BVP was solved again fifty times. The obtained mean and standard deviation of the computation times are shown in Figure 13. By taking into account all the aforementioned variables, i.e., number of nodes and the tolerable error, the Cosserat rod model is capable of achieving real-time performances of the bandwidths between 8.33 and 50 Hz.

**FIGURE 14:**
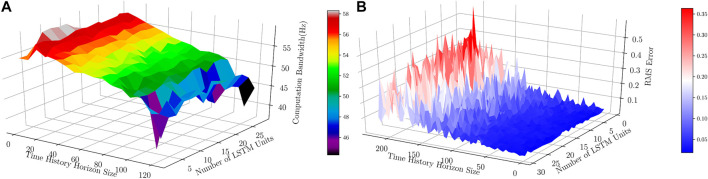
**(A)**: Computation bandwidth (Hz) obtained with respect to different number of LSTM units and size of time history horizon (for number of epochs = 25 and batch size = 1). **(B)**: RMS Error (mm) of the prediction on Data-set IV using trained models with different number of LSTM units and size of time history horizons (for number of epochs = 25 and batch size = 1).

Figure 13A shows how the computation time required for solving a solution to the BVP changes with decreasing tolerable error (*E*) and increasing percentage errors from a valid solution (at 0%), for a constant number of nodes. Also, Figure 13B shows how the computation time required for solving a solution to the BVP changes with an increasing number of nodes (*N*) and increasing percentage errors from a valid solution, for a constant tolerable error. However, It should be mentioned that the proposed RNN-based model shows a real-time performance with a bandwidth of 60.75 Hz on average for the given architecture in Figure 7, number of epochs = 25, and batch size = 1. In addition, Figure 14A demonstrates the computation bandwidth required for the prediction of the next step using the trained model with a different number of LSTM units and a different size of time history horizons in Data-set IV. The figure suggests that computation bandwidths are fairly unchanged with the number of LSTM units; however, increasing the length of time history reduces bandwidth. The optimal region maximizing the bandwidth is approximately with time history size in (2,20) and LSTM unit size in (5,15). Figure 14B suggests that RMS error of the prediction decreases by increasing the number of LSTM units and the optimal area minimizing RMS errors is approximately with time history size in (2,20) and LSTM unit size in (20,25).

To sum up, this experiment demonstrates that not only can the presented RNN-based model outperform classical modeling approaches such as the Cosserat rod model, but also it shows possibilities to use the model in practice for closed-loop control applications.

## 6 Discussion

This work suggests a distributed architecture for modeling complex dynamical systems by using multiple light-weight RNN-based models. As a result, the architecture would be easier to design and debug, and also benefits from faster convergence compared to one large network. Furthermore, large networks may take longer times to be trained, and they may not show an acceptable performance and readjusting (hyper-)parameters and restarting the training process might be necessary.

Increasing the size of history horizons in training stages may reduce the error to some extent, but on the other hand, it makes the model slower. Based on conventional dynamical models, the length of the history size should be at least 2. To reach a state-of-the-art performance, i.e., having less error and faster model simultaneously, one may prefer varied batch sizes in the training and run-time phases. As a suggestion, we can use different batch sizes for training and run-time stages. A model can be trained with appropriate batch sizes such that the model performance suits the given criteria. Afterward, one can create a new network with the pre-trained weights compiled with a batch size of 1.

The performance, i.e., the convergence and stability, of the presented algorithm in this paper, unlike conventional algorithms, is independent of the number of nodes considered for the whole manipulator. To be specific, in the analytical model, there might be a need for several discretization nodes to achieve a convergent solution with a specific tolerable error; however, in the RNN-based model, only specific points/points of interests (e.g., two actuation points in the experiment) are considered. In other words, in the experiment, 13 nodes (4 for each flexible subsection and two for each magnet, and 1 for the base) were chosen for solving the Cosserat rod model, but two nodes were selected for the RNN-based model. However, the complexity of dynamical systems (i.e., PDEs) affects the complexity of the architecture used in the RNN-based model, i.e., the number of layers and LSTM units and generally how deep the model is. Nevertheless, the suggested model suits parallel implementation and can benefit from a high bandwidth for closed-loop control applications. Furthermore, the architectures of the proposed RNN-based model can be optimized by reducing the number of layers and trainable parameters to maximize the achievable bandwidths.

The evaluations showed that incorporating poses of adjacent nodes and also wrenches as a separated input might help to have, to some extent, a generalizable model rather than just purely learning the structure of data. However, supervised learning methods likely tend to preserve structure of data, and these models might not entirely respect underlying physics (conservation laws). In other words, these methods might not be wholly physics-aware and applicable for untrained/unprecedented dynamics or geometries without any adjustment, re-training, or using techniques such as transfer learning, etc. One possible and interesting solution (Psichogios and Ungar, 1992; Lagaris et al., 1998; Raissi et al., 2019) to overcome this problem and move toward fully physics-aware neural networks is revisiting lost functions for the training process. To be specific, it is mentioned in Problem Statement Section that the idea is finding solutions for PDEs given in Eq. 1, i.e., Λ(x,t) and ϕ(x,t) for sufficiently large number (e.g., $N_{f}$) of pair $\left(x_{i},t_{i}\right)\in \left(0,L\right)\times \left[0,T\right]$ in which *L* is the unreformed length of the manipulator and parameter *T* is a user-defined time. Considering Eq. 1, a neural network can be learned by minimizing the mean squared error loss

$$\frac{1}{N_{f}}\left(\overset{N_{f}}{\underset{i=0}{\sum }}{\left\|J\omega_{t_{i}}+\omega \times J\omega +n\times \Lambda^{-1}\phi_{x_{i}}-\Lambda^{-1}\Lambda_{x_{i}}\times m-m_{x_{i}}-\Lambda^{-1}\tau \right\|}_{\left\{x_{i},t_{i}\right\}}^{2}+\overset{N_{f}}{\underset{i=0}{\sum }}{\left\|M\phi_{t_{i}t_{i}}-\Lambda \left(\Lambda^{-1}\Lambda_{x_{i}}\times n\right)-\Lambda n_{x_{i}}+f^{nc}-f\right\|}_{\left\{x_{i},t_{i}\right\}}^{2}\right)$$

This modified loss function enforces the structure imposed by Eq. 1 for large number (e.g., $N_{f}$) of pair $\left(x_{i},t_{i}\right)\in \left(0,L\right)\times \left[0,T\right]$ and the trained neural network will be aware of governing PDEs.

## 7 Conclusion

This paper describes an approach for the real-time prediction of dynamics for general continuum soft manipulators, based on machine learning techniques and Lie group variational integration methods. Poses of a soft, polymer-based manipulator, in the presence of conservative and non-conservative wrenches, are predicted and validated experimentally. The comparison results of the proposed model and a well-known model for continuum manipulators, i.e., Cosserat rod theory, are also provided, revealing the practical effectiveness of the proposed model. The presented method can be extended to different soft robots with different shapes and materials. In addition, training of physics-aware neural networks for solving PDEs and the procedure of a model-based controller design are topics of research to be studied as future work.

## Data Availability

The original contributions presented in the study are included in the article/Supplementary Material, further inquiries can be directed to the corresponding author.

## Footnotes

1 For the details see Demoures et al. (2015).

2 Dynamic equations are given in Tariverdi et al. (2020), Sec. 2.

## Appendix: Cosserat Rod Theory

The Cosserat rod model of the manipulator assigns a position $p\left(s\right)\in {\mathbb{R}}^{3}$, orientation quaternion $q\left(s\right)=\left(q_{r},q_{i}\right)\in {\mathbb{R}}^{4}$, internal force $n\left(s\right)\in {\mathbb{R}}^{3}$, and internal moment $m\left(s\right)\in {\mathbb{R}}^{3}$ to a material cross-section at centerline position s∈[0,L], where $L\in {\mathbb{R}}^{+}$ is the length of the manipulator, giving a material state vector $y={\left[p^{T},q^{T},n^{T},m^{T}\right]}^{T}$. A set of thirteen ordinary differential equations describe how the state vector evolves between centerline positions (Edelmann et al., 2017)

$$\begin{array}{l} p'=R\left(q\right)v,\\ q'=\frac{1}{2}\left[\begin{array}{c} -q_{i}^{T}\\ q_{r}I_{3}-{\left[q_{i}\right]}_{\times } \end{array}\right]R\left(q\right)u,\\ n'=-f,\\ m'=-p'\times n-\tau ,\\ v=K_{s}^{-1}R{\left(q\right)}^{T}n+\hat{v},\\ u=K_{b}^{-1}R{\left(q\right)}^{T}m+\hat{u}, \end{array}$$

where $p'\equiv \partial_{s}p$, ${\left[\cdot \right]}_{\times }:{\mathbb{R}}^{3}\to {\mathbb{R}}^{3\times 3}$ a mapping to a skew-symmetric matrix, R(q)∈SO(3) the rotation matrix associated with orientation quaternion q, $K_{s},K_{b}\in {\mathbb{R}}^{3\times 3}$ diagonal shear and bending stiffness matrices, $I_{3}\in {\mathbb{R}}^{3\times 3}$ a unit matrix, $v\left(s\right)\in {\mathbb{R}}^{3}$ and $u\left(s\right)\in {\mathbb{R}}^{3}$ the material strain and bending, and $\hat{v}\left(s\right)={\left[0,0,1\right]}^{T}$ and $\hat{u}\left(s\right)={\left[0,0,0\right]}^{T}$ the intrinsic material strain and curvature. External forces $f\left(s\right)\in {\mathbb{R}}^{3}$ and torques $\tau \left(s\right)\in {\mathbb{R}}^{3}$ determine the shape of the manipulator (Antman, 1995; Rucker and Webster, 2011). The manipulator is subject to a distributed gravity force (not shown in Figure 9) and magnetically exerted distributed forces $f_{m}$ and torques $\tau_{m}$ due to interaction of the magnets with the magnetic field $B_{m}$, where m=1,2 denotes the magnet index from the base of manipulator. Given the exerted torques and forces, the shape of the manipulator is solved as a Boundary Value Problem (BVP). The base of the manipulator is fixed to a rigid base with constant position $p_{0}$ and orientation $q_{0}$, and its distal tip is free with constant internal force $n_{L}^{d}$ and moment $m_{L}^{d}$. The proximal and distal boundary conditions are then formulated as follows, assuming no tip wrench, we have $\mathbb{P}\left(y_{0}\right)={\left[p_{0}^{T},q_{0}^{T}\right]}^{T}$ and $D\left(y_{L}\right)={\left[n_{L}^{dT},m_{L}^{dT}\right]}^{T}=0.$ The BVP is solved with a forward integration using an explicit Runge–Kutta fourth order method, and convex optimization using Levenberg–Marquardt (Till et al., 2019). The unknown proximal state parameters $\xi ={\left[n_{0}^{T},m_{0}\right]}^{T}$ are guessed and subject to the optimization where N∈ℕ is the number of discrete steps along *s*. Then $y_{N}$ are the manipulator distal state parameters obtained from the forward integration using an explicit Runge–Kutta fourth order method. The error between desired distal boundary condition $D\left(y_{L}\right)$ and $D\left(y_{N}\right)$ determines if the solution (ξ) is accepted.
